# The amoebicidal effect of *Torreya nucifera* extract on *Acanthamoeba lugdunensis*

**DOI:** 10.1371/journal.pone.0281141

**Published:** 2023-02-06

**Authors:** Min Seung Kang, Sangyoon Kim, Da Som Kim, Hak Sun Yu, Ji Eun Lee

**Affiliations:** 1 Department of Ophthalmology, Pusan National University Yangsan Hospital, Pusan National University School of Medicine, Yangsan, South Korea; 2 Research Institute for Convergence of Biomedical Science and Technology, Pusan National University Yangsan Hospital, Yangsan, South Korea; 3 Research Center of Manufacturing Process and Analytical Development, Aprogen, Osong, South Korea; 4 Department of Parasitology and Tropical Medicine, Pusan National University School of Medicine, Yangsan, South Korea; 5 Department of Ophthalmology, Pusan National University School of Medicine, Yangsan, South Korea; Xiangtan University, CHINA

## Abstract

As the number of contact lens users increases, contact lens induced corneal infection is becoming more common. *Acanthamoeba* keratitis (AK) is a type of those which is caused by *Acanthamoeba* species, and may cause severe ocular inflammation and visual loss. We evaluated whether *Torreya nucifera* (*T*. *nucifera)* extract has an anti-amoebic effect and studied its mechanism of action on *Acanthamoeba lugdunensis (A*. *lugdunensis)*. Cell viability was tested using the alamarBlue^™^ method, and the cell death mechanism was confirmed using the Tali^®^ Apoptosis Kit. The SYTOX^®^ Green assay was performed to check the plasma membrane permeability. The JC-1 dye was used to measure the mitochondrial membrane potential. A CellTiter-Glo^®^ Luminescent Assay was used to measure the adenosine-triphosphate (ATP) level. Morphological changes in the mitochondria were examined by transmission electron microscopy (TEM). Cystic changes and a decrease in cell viability after treatment with *T*. *nucifera* were observed. Both apoptotic and necrotic cells were found in the Tali^®^ Apoptosis assay. There was no significant difference in plasma membrane permeability between the control and *T*. *nucifera* treated groups. The collapse of the mitochondrial membrane potential and reduced ATP level in *A*. *lugdunensis* was confirmed in the groups treated with *T*. *nucifera*. Structural damage to the mitochondria was observed on TEM in the groups treated with *T*. *nucifera*. *T*. *nucifera* showed an anti-amoebic effect on *A*. *lugdunensis*, by inducing the loss of mitochondrial membrane potential. Thus, it could be a future therapeutic agent for AK.

## Introduction

*Acanthamoeba* species causes a vision-threatening corneal infection known as *Acanthamoeba* keratitis (AK). Moreover, the increase in the number of contact lens (CL) users and the lack of proper hygiene habits and poor handling procedures can lead to an increased risk of infections, including AK [1, 2]. Since AK is a serious ocular infectious disease that can cause loss of vision, timely diagnosis and effective treatment at the early stage of the infection are very important. Some antiseptics such as polyhexamethylene biguanide (PHMB) and chlorhexidine 0.02% are widely used, and known to be effective against the trophozoites and cysts of *Acanthamoeba* [1, 2]. However, in many cases, the therapeutic effect is limited, and the occurrence of corneal opacity and corneal toxicity has been reported. Hence, research for the development of new therapeutic agents for this condition is ongoing. The pathogenesis and cellular differentiation processes in *Acanthamoeba* are still not completely understood, and there is an urgent need for further investigation.

*Torreya nucifera (T*. *nucifera)* is an evergreen conifer growing in Korea, China, and Southern Japan. It has been widely used as a traditional medicine due to its pharmacological effects on constipation, diabetes mellitus, and hemorrhoids [3, 4]. In addition, it has been reported that *T*. *nucifera* leaves showed an anti-inflammatory effect and exhibited inhibitory activity on the main protease of coronavirus that causes the severe acute respiratory syndrome [5, 6]. Although *T*. *nucifera* has been used as a traditional anti-inflammatory agent, there are no reports about its effect on the treatment of parasites.

In this study, we aimed to determine whether *T*. *nucifera* has an anti-amoebic effect. In addition, we aimed to elucidate the cellular biological mechanism of action of *T*. *nucifera* extract on *Acanthamoeba lugdunensis (A*. *lugdunensis)* to explore its use as a new therapeutic agent for AK.

## Materials and methods

### Source of *T*. *nucifera*

*T*. *nucifera* was obtained from Durae Corporation (Gunpo, Korea). It was dissolved in Dimethyl sulfoxide (DMSO) and diluted with the culture medium to obtain the desired concentrations. The control group was treated only with DMSO, and the final concentration of DMSO was equal between the *T*. *nucifera*-treated and control groups.

### Preparation of *Acanthamoeba*

Although *Acanthamoeba castellanii* and *Acanthamoeba polyphaga* are common causative agents of AK in many countries [7–9], this study examined *A*. *lugdunensis*, the most frequently isolated type of *Acanthamoeba*, from contact lens storage cases in Korea [10–12]. The specimen of *A*. *lugdunensis* was obtained from a patient with AK before starting medication, and was identified by riboprinting and 18S rDNA sequence analyses as previously described [9, 13–15]. *A*. *lugdunensis* trophozoites were cultured in a culture flask (Falcon: BD Biosciences; Franklin Lakes, New Jersey, USA) with peptone–yeast extract/glucose (PYG) medium (20.02 g of Bacto Proteose Peptone and 1.00 g of yeast extract in 950 mL of pure water, 50.0 mL of 2 M D(+)glucose, 10.0 mL of 0.4 M Magnesium sulfate heptahydrate (MgSO4·7H2O), 8.0 mL of 0.05 M Calcium chloride (CaCl2), 34.0 mL of 0.1 M sodium citrate 2H2O, 10.0 mL of 0.005 M Ferrous Ammonium Sulfate Hexahydrate ((Fe(NH4)2(SO4)2·6H2O), 10.0 mL of 0.25 M Sodium phosphate dibasic heptahydrate (NaHPO4·7H2O), and 10.0 mL of 0.25 M Potassium dihydrogen phosphate (KH2PO4)) at room temperature. Prior to the experiment, the medium was changed by vigorously shaking the culture bottles, removing the medium, and adding the new medium. For the experiments, the number of *A*. *lugdunensis* applied to a substrate was determined by counting the trophozoites in advance using a Neubauer hemocytometer.

### *In vitro* effect on the trophozoite stage of *A*. *lugdunensis*

The anti-*Acanthamoeba* effect of *T*. *nucifera* was analyzed using the alamarBlue^™^ assay (Life Technologies; Carlsbad, California, USA). Briefly, *A*. *lugdunensis* were seeded in duplicate on a 96-well microtiter plate from a stock solution of 1 X 10^4^ cells/mL. Ten μL of 25 μg/mL and 50 μg/mL *T*. *nucifera* solutions were prepared and added into each different well except the control group. Finally, 10 μL of the alamarBlue^™^ Reagent was added to each well. Plates were incubated for 96 h at 26°C with slight agitation. Subsequently, the plates were analyzed with a microplate reader (PerkinElmer; Waltham, Massachusetts, USA) using the emitted fluorescence (570 nm).

### Image-based cytometry assays for the determination of cell death

Annexin-V/propidium iodide (PI) double-stain apoptosis detection kit (Tali^®^ Apoptosis Kit—Annexin V Alexa Fluor^®^ 488 & Propidium Iodide) and Tali^®^ Image-Based Cytometer (Life Technologies) were used according to the manufacturer’s instructions. *A*. *lugdunensis* was seeded in duplicate on a 24-well μL plate from a stock solution of 1 X 10^5^ cells/mL. One hundred μL of 25 μg/mL and 50 μg/mL *T*. *nucifera* solutions were prepared and added into each different well except the control group. Cells were incubated for 24 h, and centrifuged at 1500 rpm for 10 min. They were washed twice with Annexin Binding Buffer and incubated with 5 μL of annexin-V for 20 min and centrifuged at 1500 rpm for 10 min. They were then incubated for 3 min at room temperature in 1 μL of PI. Finally, the cells were loaded into a Tali^®^ Cellular Analysis Slide and analyzed using the Tali^®^ Image-Based Cytometer. Data were collected using the Tali^®^ data acquisition and analysis software.

### Plasma membrane permeability

The SYTOX^®^ Green assay (Thermo Fisher; Waltham, Massachusetts, USA) was performed according to the manufacturer’s instructions to detect the membrane permeability of *A*. *lugdunensis* alterations. A total volume of 90 μL of *A*. *lugdunensis* were seeded in duplicate on a 96-well black wall microtiter plate from a stock solution of 1 X 10^4^ cells/mL. Twentyfive μg/mL and 50 μg/mL *T*. *nucifera* solutions were prepared and added into each different well (a negative control with PYG and positive control with 2.5% of Triton X-100 [Sigma Aldrich; St. Louis, Missouri, USA]), as previously described [16–18]. After incubation for 24 h at 26°C, SYTOX Green reagent was added at a final concentration of 1 μM for 15 min under dark conditions. Cells were observed using an EnSpire microplate reader (PerkinElmer) with an excitation wavelength of 504 nm and an emission wavelength of 523 nm.

### Changes in the mitochondrial membrane potential

The mitochondrial membrane potential was measured using the JC-1 mitochondrial membrane potential detection kit (Cayman Chemical; Ann Arbor, Michigan, USA). The JC-1, a lipophilic cationic probe, accumulates in the mitochondrial matrix based on the membrane potential. *A*. *lugdunensis* (900 μL) were seeded in duplicate on a 24-well microtiter plate from a stock solution of 1 X 10^5^ cells/mL. Twentyfive μg/mL and 50 μg/mL *T*. *nucifera* solutions were prepared and added into each different well except the control group. After incubation for 24 h at 26°C, the cells were incubated for 24 h and centrifuged at 1500 rpm for 10 min, and then washed and resuspended in JC-1 buffer twice. The cell pellet was mixed with 100 μL buffer, and 10 μL of JC-1 was added. After incubation for 30 min at 26°C, the cells were centrifuged at 1500 rpm for 10 min. The cells were then analyzed using confocal fluorescence measurement.

### Measurement of ATP

ATP level was measured using a CellTiter-Glo^®^ Luminescent Cell Viability Assay (Promega; Medison, Wisconsin, USA), according to the manufacturer’s instructions. To confirm the effect of *T*. *nucifera* on the intracellular ATP production, *A*. *lugdunensis* were seeded in duplicate on a 24-well microtiter plate from a stock solution of 1 X 10^4^ cells/mL. Ten μL of 25 μg/mL and 50 μg/mL *T*. *nucifera* solutions were prepared and added into each different well except the control group. After incubation for 24 h at 26°C, 100 μL of CellTiter-Glo^®^ was added to each well, and the plates were shaken for 2 min to induce cell lysis. After 10 min of incubation at room temperature, the luminescence was measured using the EnSpire microplate reader.

### Morphologic assay of the mitochondria

For transmission electron microscopy, the *A*. *lugdunensis* that had been grown to confluence in 24-well plates were incubated in the Dulbecco Modified Eagle Medium containing *T*. *nucifera* extract at a concentration of 50 ug/mL and phosphate buffer control for 18 h under 5% CO_2_ at 37°C. The *A*. *lugdunensis* were incubated at 37°C for 24 h after rinsing with PBS. The *A*. *lugdunensis* were fixed with 2.5% glutaraldehyde in 0.1 mol/L phosphate buffer (pH 7.4) for 12 h and postfixed with 0.1% osmium tetroxide for 2 h. After rinsing with 0.1 mol/L of phosphate buffer and dehydrating in a graded series of ethanol, the specimens were embedded in an Epon 812 mixture. An ultrathin section of 60–80 nm was cut, stained with uranyl acetate and lead citrate, and examined by transmission electron microscopy (JEOL1200EX; Jeol, Tokyo, Japan).

### Statistical analysis

All experiments were performed five times, and the results are expressed as the means ± SD (S1 Table). The data were analyzed using GraphPad Prism 5.0 software (GraphPad Software Inc.; La Jolla, California, USA). For comparisons of the viability *of A*. *lugdunensis*, plasma membrane permeability, and ATP levels between the groups, an unpaired *t*-test was used. A *P*-value < 0.05 was considered statistically significant.

## Results

### *In vitro* effect on the trophozoite stage of *A*. *lugdunensis*

**Fig 1:**
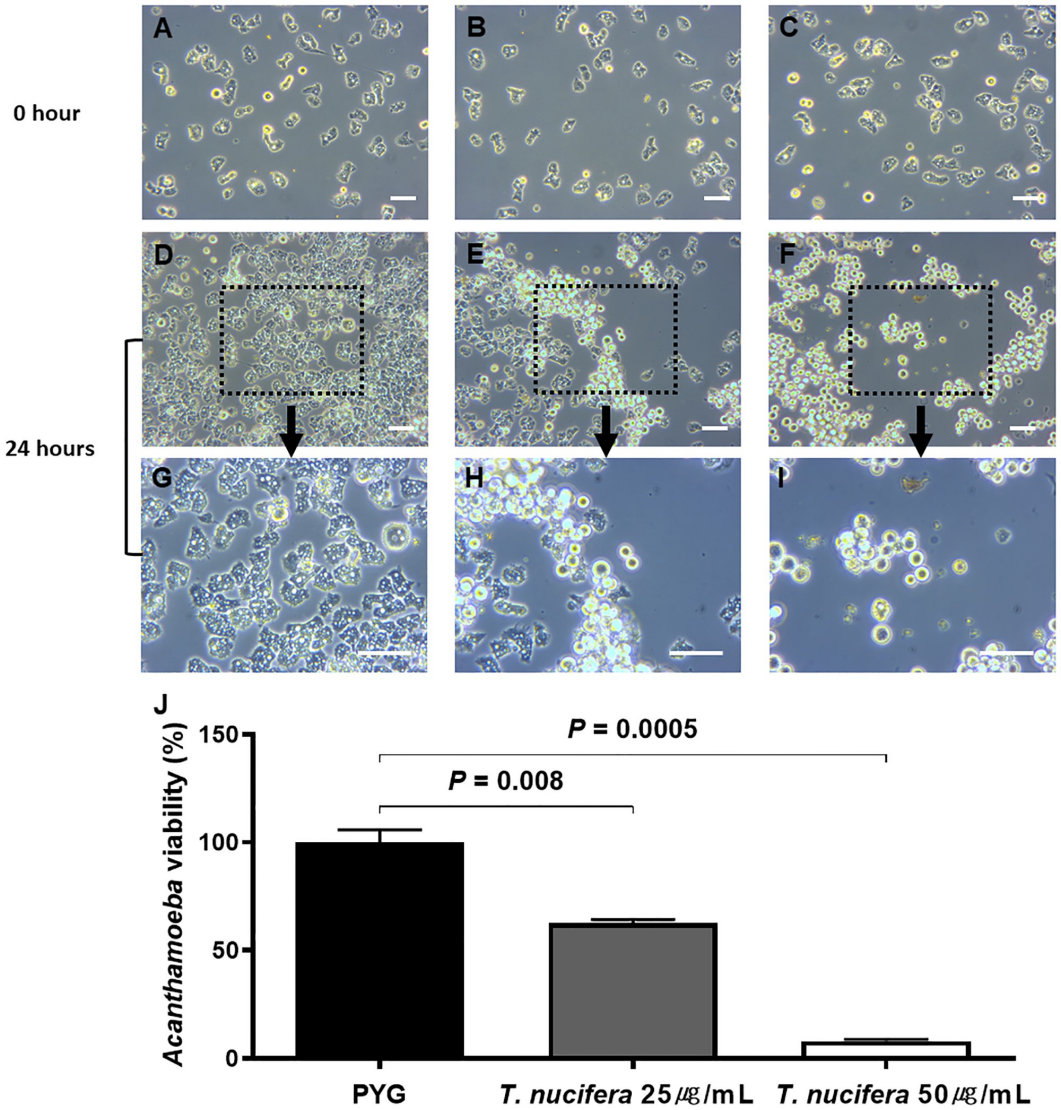
Morphological changes and cell viability in *A*. *lugdunensis* after 24 hours of treatment with *Torreya nucifera*. Compared with the control group (A, D, G), groups treated with 25 μg/mL (B, E, H) and 50 μg/mL (C, F, I) of *T*. *nucifera* showed morphological changes in which encystation from trophozoites to cysts and shrunken and unviable cells were observed. Cell viability was significantly decreased in the *T*. *nucifera treated* group and was dose-dependent (J). The scale bar represents 50 μm..

To confirm whether *T*. *nucifera* had an anti-amoebic effect, we analyzed the morphological changes in *A*. *lugdunensis* after treatment with 25 μg/mL and 50 μg/mL of *T*. *nucifera* compared with treatment with the control group. The groups treated with 25 μg/mL and 50 μg/mL of *T*. *nucifera* showed a morphological change during which was encystation from the trophozoites was observed. It was also confirmed that there was a concentration dependence; higher encystation was observed in the group treated with 50 μg/mL of *T*. *nucifera* than in that treated with 25 μg/mL (Fig 1A–1I). In addition, compared with the control group, which was treated with PYG medium, the group treated with 50 μg/mL of *T*. *nucifera* demonstrated a significant decrease in viability, and the amoebicidal activity was dose-dependent (Fig 1J).

### Image-based cytometry assays for the determination of cell death

**Fig 2:**
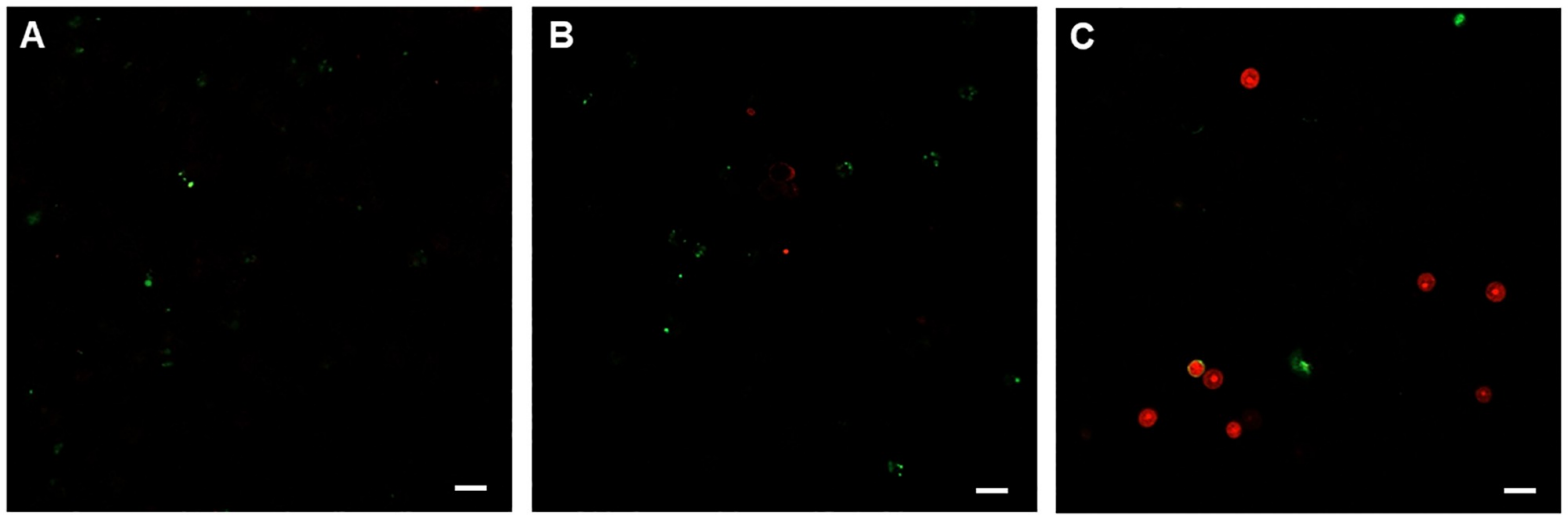
Image-based cytometer analysis to determine cell death after 24 hours of treatment with *Torreya nucifera*. The process of cell death was evaluated using the Tali^™^ Image-based Cytometer using the Tali^™^ apoptosis kit. In the groups treated with 25 μg/mL (B) and 50 μg/mL (C) of *T*. *nucifera*, the green and red fluorescence indicating apoptosis and necrosis, respectively were clearly observed unlike that in the control group (A). The scale bar represents 20 μm.

To demonstrate the pathways of cell death of *A*. *lugdunensis* following the treatment with *T*. *nucifera*, Tali^®^ Apoptosis Kit and Tali^®^ Image-Based Cytometer were used. In the group treated with 25 μg/mL of *T*. *nucifera*, faint green and red fluorescence was observed, while cysts were clearly observed in the group treated with 50 μg/mL *T*. *nucifera*. We confirmed the necrosis as well as apoptosis of *A*. *lugdunensis* following treatment with a 50 μg/mL concentration of *T*. *nucifera* extract for 24 hours (Fig 2).

### Plasma membrane permeability

**Fig 3:**
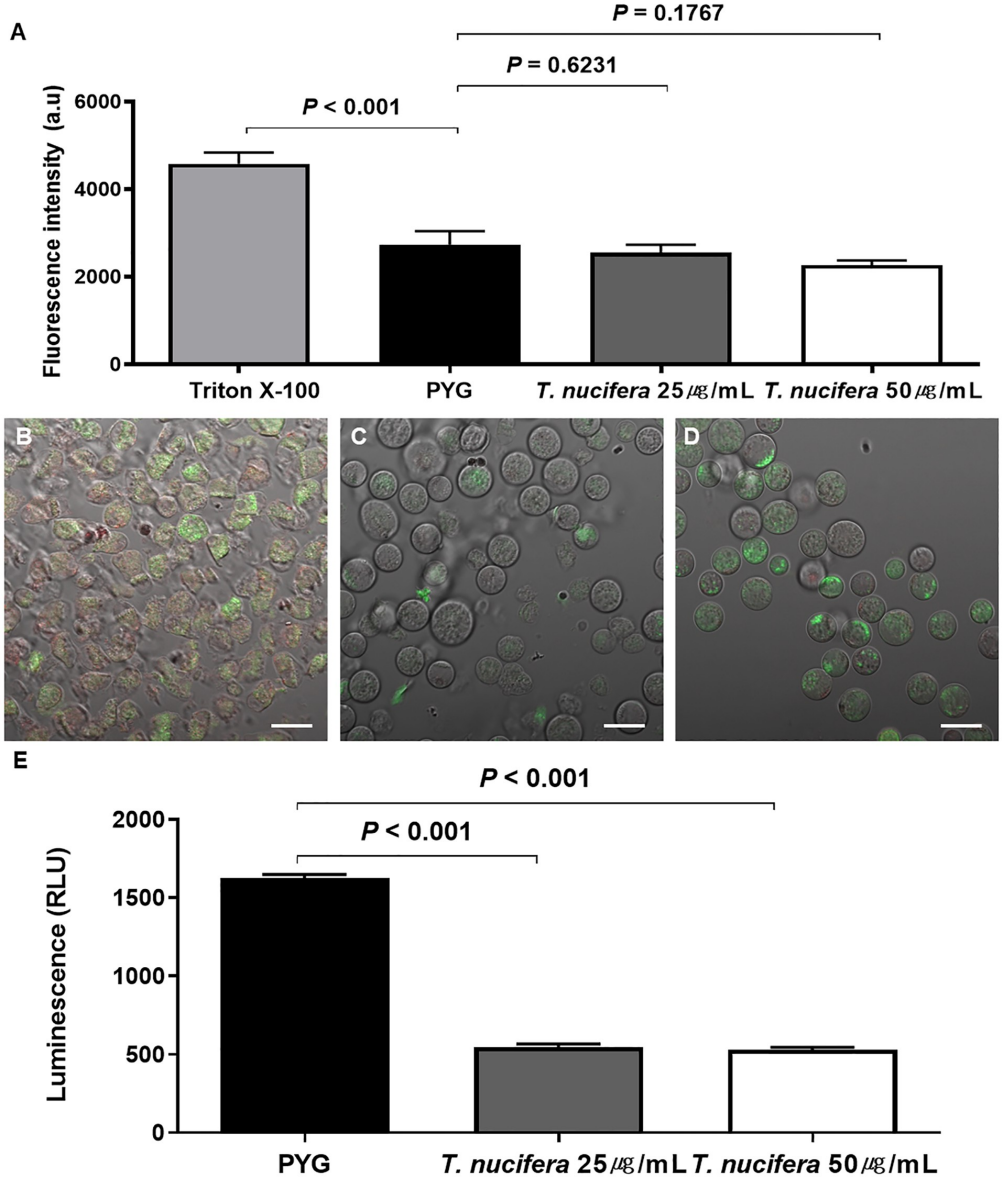
Cell membrane Permeability changes, mitochondrial membrane potential changes, and the effect of *Torreya nucifera* on ATP production in *A*. *lugdunensis*. Unlike the positive control group (Triton X-100), which showed high fluorescence, the groups treated with 25 μg/mL and 50 μg/mL of *T*. *nucifera* showed significantly low fluorescence similar to that seen in the negative control group (A). In the control group, the mitochondrial membrane potential gradient was normal (B). Groups treated with 25 μg/mL (C) and 50 μg/mL (D) of *T*. *nucifera* showed the collapse of the mitochondrial membrane potential gradient indicated by the green fluorescence in the cytoplasm. The scale bar represents 20 μm. ATP production showed that the luminescence amount was significantly reduced in the groups treated with 25 μg/mL and 50 μg/mL of *T*. *nucifera* compared to that in the control group (E).

The SYTOX Green assay was performed to detect changes in *A*. *lugdunensis* membrane permeability since it is an important indicator of cell health and the process of cell death. In the positive control group treated with 2.5% Triton X-100, high fluorescence was seen in the EnSpire microplate reader because of severe plasma membrane damage. Unlike the positive control group, the negative control group treated with PYG medium and the experimental groups treated with 25 μg/mL and 50 μg/mL of *T*. *nucifera* showed a relatively less significant fluorescence. There was no significant difference in the fluorescence between the negative control group and *T*. *nucifera* treated groups (Fig 3A).

### Changes in the mitochondrial membrane potential

The use of JC-1 Mitochondrial Membrane Potential Assay Kit helped us to detect the effect of *T*. *nucifera* on the mitochondrial membrane potential of *A*. *lugdunensis*. In the control group treated with the PYG medium, the mitochondrial membrane potential gradient was normal, and red fluorescence was observed in the mitochondrial matrix. However, in the experimental groups treated with 25 μg/mL and 50 μg/mL of *T*. *nucifera*, the membrane potential of the mitochondria decreased, and it was difficult for the JC-1 dye to enter the mitochondrial matrix. The green fluorescence was observed in the cytoplasm, and the intensity of the fluorescence was higher in the group treated with 50 μg/mL than in that treated with 25 μg/mL of *T*. *nucifera*. The change in the gradient of mitochondrial membrane potential depended on the concentration of the *T*. *nucifera* (Fig 3B–3D).

### Measurement of ATP

The normal mitochondrial functioning depends on suitable mitochondrial membrane potential, as well as an appropriate level of ATP. To confirm whether *T*. *nucifera* affects the *A*. *lugdunensis* ATP levels, CellTiter-Glo was used to quantify ATP. In the control group treated with PYG medium, the luminescence amount was 1624 Relative light units (RLU). However, the luminescence amount was significantly reduced by approximately one-third in the 25 μg/mL (547.8 RLU) and 50 μg/mL (531.4 RLU) *T*. *nucifera* treatment groups. Thus, it was confirmed that the ATP levels of *A*. *lugdunensis* significantly decreased following treatment with *T*. *nucifera* extract for 24 hours (Fig 3E).

### Mitochondrial morphologic assay

**Fig 4:**
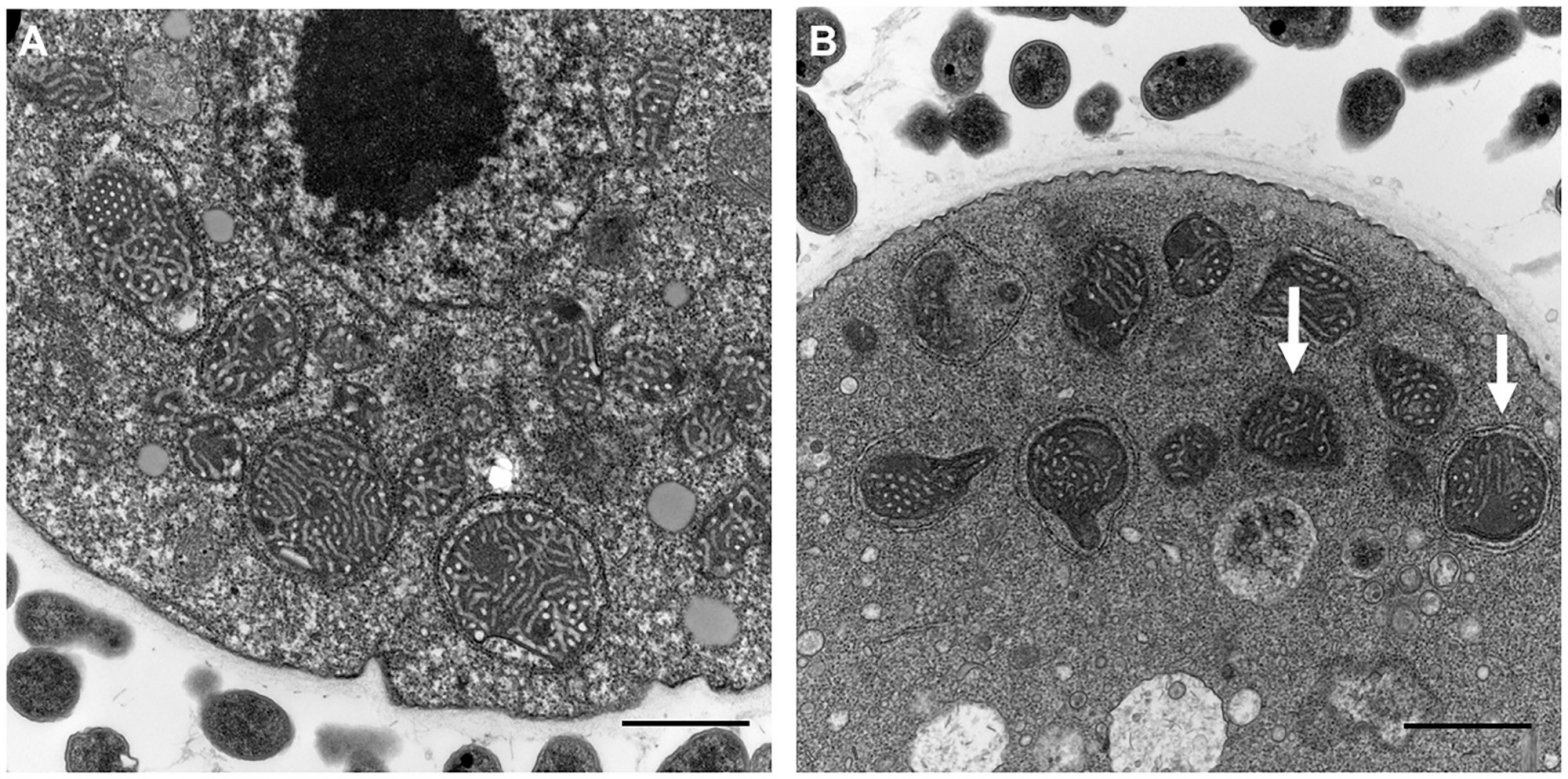
Mitochondrial morphologic changes in *A*. *lugdunensis*. After treatment with 50 μg/mL (B) of *T*. *nucifera*, structural damage and decreased wrinkling was observed compared with that in the control group (A). The scale bar represents 1 μm.

Transmission electron microscopy was used to confirm the morphological change in the mitochondria of *A*. *lugdunensis*. It was confirmed that the mitochondria showed decreased wrinkling and structural damages compared to those of the control group treated with PYG medium (Fig 4).

## Discussion

The number of reported cases of AK has increased due to the increased use of contact lenses. Increased awareness combined with early diagnosis of the disease is important for better outcomes [1, 2]. However, the pathogenesis and cellular differentiation processes of *Acanthamoeba* are not fully understood; they hold the key to improved diagnosis and the development of effective therapeutic approaches. In recent times, there has been some research on the treatment of *Acanthamoeba* infection to elucidate and select new therapeutic targets [1]. *T*. *nucifera* is an evergreen tree that grows in Korea and Japan, and its seeds, leaves, and stems have long been used as edible products and herbal medicines in Korea. Some previous reports demonstrated the pharmacological activity, including antioxidant, anti-inflammatory, hepatoprotective, and neuroprotective properties of *T*. *nucifera* [4, 19–21]. However, the effect of *T*. *nucifera* on *Acanthamoeba* has not been reported, although it has traditionally been used as anthelmintic for extermination of parasitic worms in Japan [22]. In this study, we conducted experiments to show the anti-amoebic effect and elucidate the mechanism of action of *T*. *nucifera* on *A*. *lugdunensis*.

We confirmed the morphological changes in *A*. *lugdunensis* following the treatment with *T*. *nucifera* extract, such as encystation from trophozoites to cysts and shrunken or unviable cells. The amoebicidal cell viability test showed a significant dose-dependent decrease in the number of *A*. *lugdunensis* cells in the *T*. *nucifera* treated group. The morphological changes and decrease in cell viability might be because of the amoebicidal effect *of T*. *nucifera* extract. We further verified whether *A*. *lugdunensis* cell death occurs due to *T*. *nucifera*, and found that *T*. *nucifera* induced both apoptosis and necrosis of amoebic cysts. Programmed cell death is a type of self-destruction of the cells to eliminate the damage in the cells [23]. There are several types of programmed cell deaths, such as apoptosis, autophagy, and necrosis [24]. The process of programmed cell death shares several features in multicellular organisms, including chromatin condensation, cell shrinkage, and loss of mitochondrial membrane potential [25].

**Fig 5:**
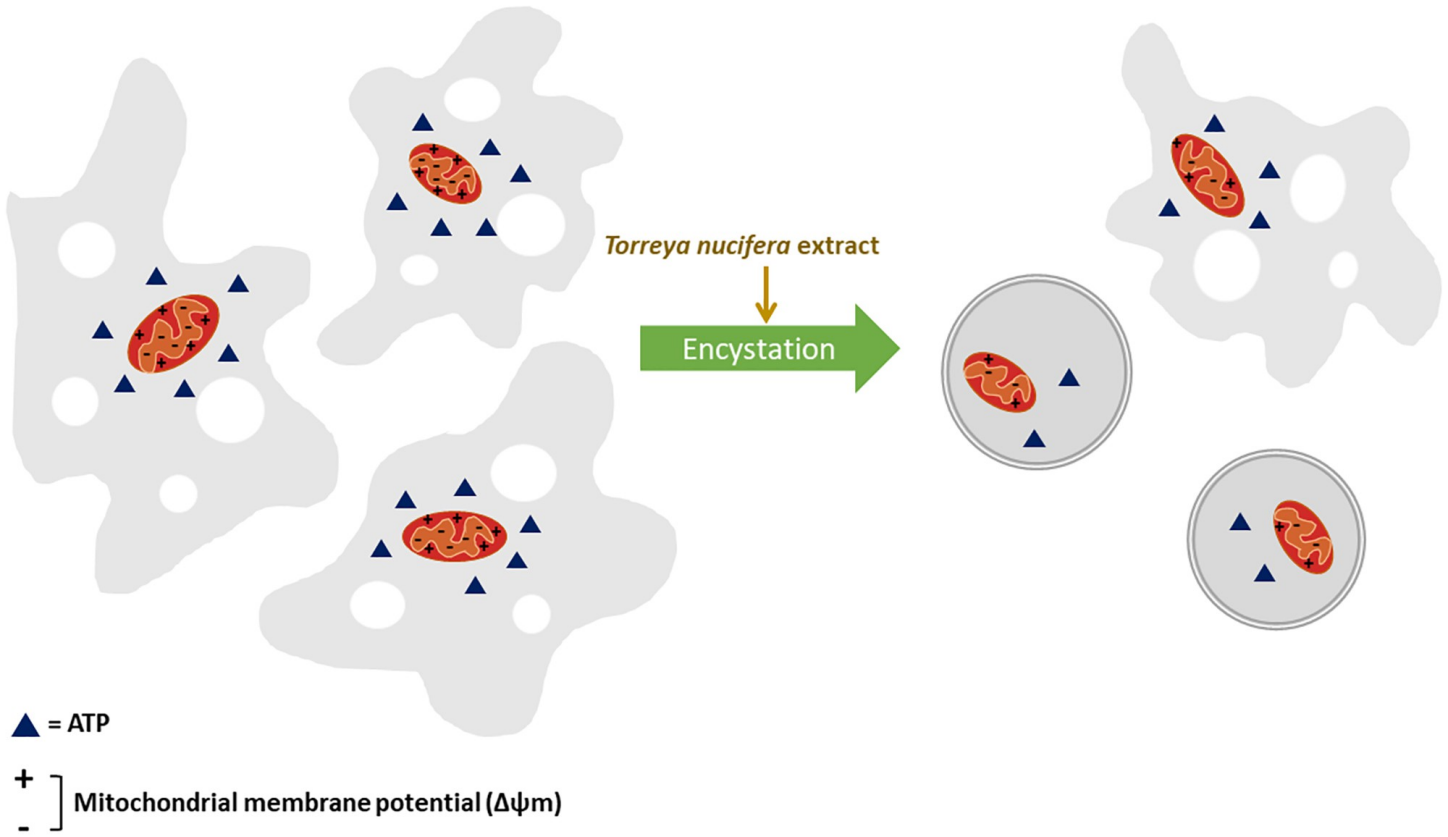
Model diagram of the cellular biological mechanism of the amoebicidal effect of *Torreya nucifera*. When *A*. *lugdunensis* were treated with *T*. *nucifera*, the morphological change of encystation was confirmed. The decrease in mitochondrial ATP level and collapse of the mitochondrial membrane potential is the likely amoebicidal mechanism of *T*. *nucifera*.

We tried to confirm the functional state of mitochondria through ATP level quantification, changes in the mitochondria membrane potential, and plasma membrane permeability to understand the mechanism of amoebic cell death after treatment with *T*. *nucifera* extract. There was no significant difference in the plasma membrane permeability compared to that in the control group; however, a significant difference was observed in the ATP level and changes in the membrane potential after *T*. *nucifera* treatment. One of the indicators of the cell death process is the loss of mitochondrial membrane potential; nevertheless, the process of cell death requires energy in the form of ATP to initiate and support all the mechanisms involved in this process. Though treatment with *T*. *nucifera* extract did not affect the membrane permeability of *A*. *lugdunensis*, it affected the mitochondrial membrane potential, resulting in loss of mitochondrial function, thereby reducing the ATP levels. Hence, it can be inferred that the *T*. *nucifera* extract inhibited the activity and led to the death of *A*. *lugdunensis* (Fig 5).

Transmission electron microscopy showed structural changes in the mitochondria, such as decreased wrinkling and structural damage. Our results clearly demonstrated that the mechanism of cell death of *A*. *lugdunensis* was due to the changes in the mitochondrial membrane potential through mitochondrial structural damage rather than due to the damage to the plasma membrane.

This study had some limitations. First, there are no previous studies that have evaluated the anti-amoebic effect of *T*. *nucifera* with which our results can be compared. Second, this study did not determine the minimum inhibitory concentration of *T*. *nucifera* extract on *A*. *lugdunensis*; hence, further studies are necessary. Finally, this study examined only the *in vitro* effects of *T*. *nucifera* extract on *A*. *lugdunensis*. Further studies on animal models should be conducted before considering its use in humans.

## Conclusions

*T*. *nucifera* could be a useful future therapeutic agent for treating *A*. *lugdunensis* infection. Although detailed *in vivo* studies and clinical trials are necessary to evaluate the use of *T*. *nucifera* in the treatment of *A*. *lugdunensis* infection in the clinical setting, the present study is an interesting starting point for further studies to develop a novel and effective treatment against AK using *T*. *nucifera*.

## Supporting information

S1 Table Dataset yielded from the experiment. The dataset is the result from alamarBlue^™^ assay, ATP measurement by CellTiter-Glo^®^ Luminescent Cell Viability Assay, and membrane permeability measurement by the SYTOX Green assay, which were conducted in this study.
(XLSX)
