# Correction: The amoebicidal effect of *Torreya nucifera* extract on *Acanthamoeba lugdunensis*

**DOI:** 10.1371/journal.pone.0294194

**Published:** 2023-11-03

**Authors:** Min Seung Kang, Sangyoon Kim, Da Som Kim, Hak Sun Yu, Ji Eun Lee

There are errors in the Funding section. The correct Funding statement is: This work was supported by funding provided by National Research Foundation funded by the Ministry of Science, Information and Communication Technology (NRF2020R1A2C10099751) and 2022 research grant from Pusan National University Yangsan Hospital.
